# The Occurrence of Zearalenone in South Korean Feedstuffs between 2009 and 2016

**DOI:** 10.3390/toxins9070223

**Published:** 2017-07-15

**Authors:** Hansub Chang, Woori Kim, Ju-Hee Park, Dongho Kim, Choong-Ryeol Kim, Soohyun Chung, Chan Lee

**Affiliations:** 1 National Agricultural Products Quality Management Service, 141, Yongjeon-ro, Gimcheon-si 39660, Gyeongsangbuk-do, Korea; jjhs@korea.kr (H.C.); anoldmu@korea.kr (D.K.); kimchr@korea.kr (C.-R.K.); 2 Advanced Food Safety Research Group, BrainKorea21 Plus, Department of Food Science and Technology, Chung-Ang University, Anseong-si 17546, Gyeonggi-do, Korea; dnflvkxld123@naver.com (W.K.); bjhwngml@naver.com (J.-H.P.); 3 Department of Integrated Biomedical and Life Science, Korea University, Seoul 02841, Korea; chungs59@korea.ac.kr

**Keywords:** mycotoxin, zearalenone, feedstuffs

## Abstract

Mycotoxins produced by *Fusarium* plant pathogen species have harmful effects on humans and livestock by natural contamination in food and feed. Zearalenone, one of the well-known *Fusarium* mycotoxins, causes hyperestrogenism and toxicosis resulting in reproductive dysfunction in animals. This study investigated the occurrence of zearalenone in feedstuffs (compound feeds, feed ingredients) between 2009 and 2016 in South Korea to obtain information on zearalenone contamination in feeds for management. A total of 653 animal feed samples (494 compound feeds, 159 feed ingredients) produced domestically were sampled five times from 2009 to 2016 (2009, 2010, 2012, 2014, and 2016) from feed factories in South Korea. The levels of zearalenone were analyzed every year by high-performance liquid chromatography (HPLC) after pretreatment with an immunoaffinity column showing limit of detection (LOD) and limit of quantification (LOQ) of 0.1–3 μg/kg and 0.3–8 μg/kg, respectively. Four feed samples out of 494 compound feeds exceeded the EU and South Korea commission regulations over the eight-year test period, and no feed ingredients exceeded the guidelines.

## 1. Introduction

**Figure 1:**
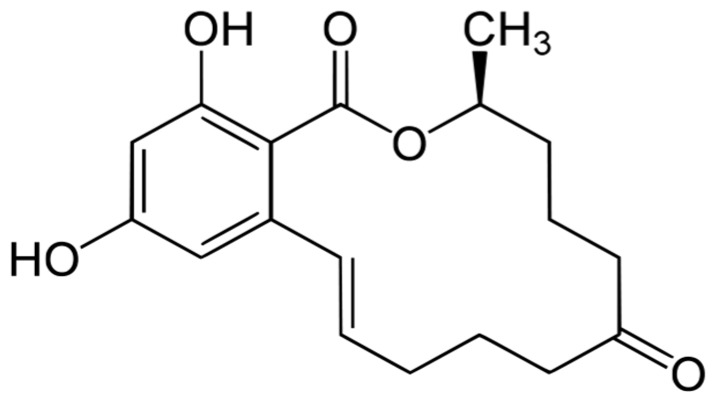
Chemical structure of zearalenone (ZEN).

Mycotoxins are secondary metabolites produced by fungi such as *Fusarium*, *Aspergillus*, or *Penicillium* genera during crop cultivation or storage processes [1]. Mycotoxins have been increasingly mentioned worldwide as an important issue because of their acute and chronic toxicity to humans and animals [2]. Many *Fusarium* mycotoxins, including deoxynivalenol (DON), zearalenone (ZEN), and fumonisin (FUM), are distributed widely in food and animal feed and can cause different diseases in humans, animals, and even in plants [3]. One of the well-known *Fusarium* mycotoxins, zearalenone (ZEN), is a white crystal granule exhibiting a melting point of 164~165 °C and a maximum absorbance at 264 nm in UV-absorption. ZEN is insoluble in water due to its hydrophobicity, but is soluble in water-soluble alkali and other organic solvents. The level of ZEN in crops is not reduced during storage or processing at high temperature because of its heat stability [4]. The chemical structure of ZEN is shown in Figure 1.

ZEN causes hyperestrogenism and toxicosis resulting in reproductive dysfunction in animals because it competitively binds to the mammalian estrogen acceptor. Pigs are reported as the most susceptible animal to ZEN among livestock [5]. Acute toxicity of ZEN by sub-acute exposure is relatively low, and its lethal dose was reported as over 4000 mg/kg (b.w.). Due to its low toxicity, the International Agency for Research on Cancer (IARC) classified ZEN in group 3 (not classifiable as carcinogenic to humans) [6].

Grains, grain byproducts, and vegetable proteins found in animal feed are major nutrient sources for the growth of fungi [7]; therefore, mycotoxins produced by fungi in animal feeds are an important management issue in feed safety. In Korea, 18,640 tons of compound feed were produced domestically in 2012, and 15,350 tons of feed ingredients were imported in the same year from major exporting countries such as the USA, Canada, Europe, China, South Africa, South-East Asia, India, and Australia. This variety of feed ingredients from different countries leads to difficulty in controlling mycotoxin levels in compound feeds.

In Turkey, cattle feeds were contaminated with ZEN in 45.2% of tested samples [8], and feed ingredients were contaminated with ZEN in 31.58% of total samples [9]. In Slovakia, 88% of tested poultry feed samples were contaminated with ZEN in 2004 [10]. In Asia and Oceania, Binder et al. reported mean ZEN contamination level of 0.077 mg/kg in feed ingredients [11]; in Korea, ZEN was detected in 98% of tested feeds, with concentrations ranging from 0.009 to 0.405 mg/kg [12].

The European Commission (EU) has suggested guidelines for maximum aflatoxin, ochratoxin, or other *Fusarium* mycotoxin levels in animal feeds, and the USA has also defined guidelines for aflatoxin (AFT), deoxynivalenol (DON), and fumonisin (FUM) in feeds [13]. So far, the levels of AFT and OCT in animal feeds have been controlled according to the guidelines for livestock control and fish feed acts in South Korea; recently, the guidelines for *Fusarium* mycotoxins were set based on the monitoring results and guidelines of the European Union (EU). In this study, data on the contamination levels of ZEN in animal feeds that were collected for many years are reported, and the contamination risk of ZEN in animal feeds in Korea is discussed. The guidelines for ZEN in the EU and South Korea are shown in Table 1.

## 2. Results

### 2.1. Method Validation

**Figure 2:**
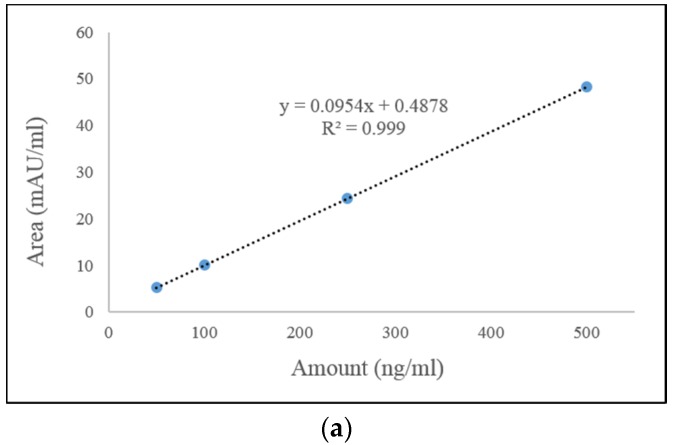

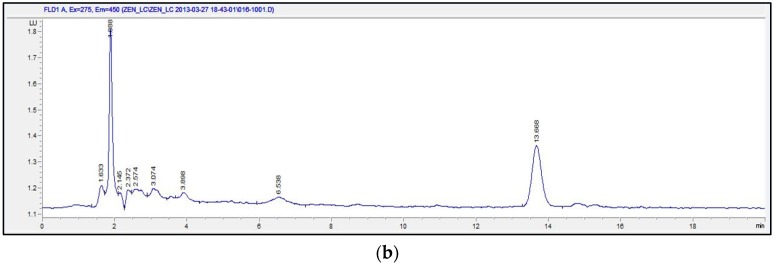
Calibration curve for ZEN (**A**) and HPLC chromatogram (**B**). *R*^2^: Coefficient of determination.

**Figure 3:**
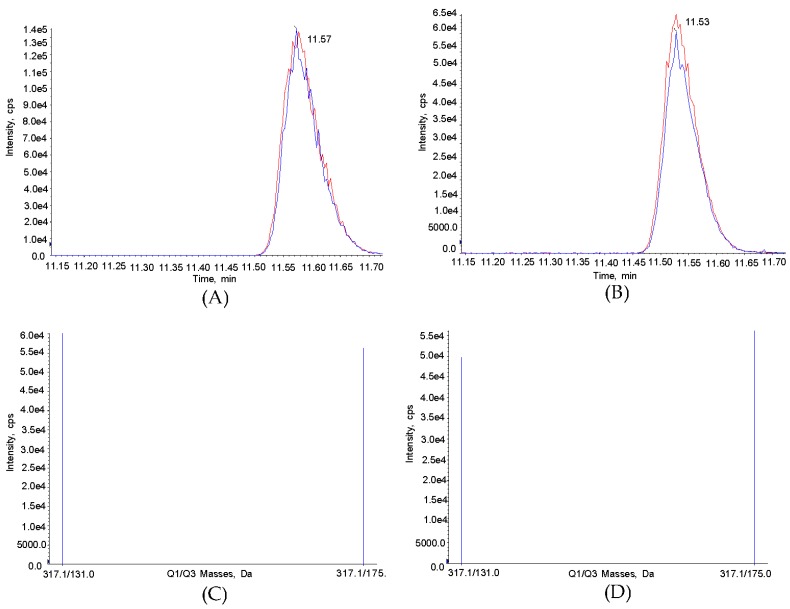
Identification of ZEN by LC-MS/MS. Extracted ion chromatogram of standard ZEN (75 ppm) (**A**) and ZEN in sample (**B**); Ion spectrum of standard ZEN (**C**) and ZEN in sample (**D**).

HPLC chromatogram analysis clearly demonstrated that ZEN was well separated from any potential interference due to the pretreatment with Immuno Afiinity Column (IAC), and no interfering peaks were observed during analysis. The limit of detection (LOD) and limit of quantitation (LOQ) for ZEN were estimated as 0.1~3 μg/kg and 0.3~8 μg/kg, respectively. The coefficient of determination in the solvent based standard curve for ZEN was calculated as over 0.999 using ZEN as a standard reference. The components of most tested feeds were based on corns, soybeans, and wheat, and the ratio of composition was different according to type of animal. In general, pig feeds mainly contain the major components in an average composition ratio among tested feeds. Therefore, a ZEN-free pig feed was applied as a blank sample for further experiments. The average recovery ratio of ZEN was 83~111%, and relative standard deviation (RSD) was calculated as 1.4~14.7%, which meets the commission regulation for ZEN in the EU (70~120% of accuracy, under 20% of precision) [14]. The calibration curve and detailed data are shown in Figure 2 and Table 2. After analysis of ZEN in HPLC chromatogram, the peak was further identified by LC-MS analysis with its extracted ion chromatogram (XIC) and mass spectrum as shown in Figure 3. Extracted ion chromatograms of ZEN in standard solution (A) and in sample (B) exhibited the same precursor ion (*m*/*z* 317.1 [M − H]^−^) (B); and two product ions (*m*/*z* 131.0, *m*/*z* 175.0) from the precursor ion of ZEN (C) matched exactly with those from sample (D).

### 2.2. Occurrence of ZEN in Compound Feeds between 2009 and 2016

ZEN contamination levels were evaluated from 494 compound feed samples (174 cattle feeds, 160 pig feeds, and 160 poultry feeds) collected in 2009, 2010, 2012, 2014, and 2016. ZEN was detected in 97.7% of cattle feeds, 95.0% of pig feeds, and 96.3% of poultry feeds. In total, 96.4% of samples were contaminated with ZEN. The ZEN concentrations in total feeds ranged from 1 to 932 μg/kg with a mean value of 70 μg/kg. The detailed data is shown in Table 3.

In a one-way layout-dispersion analysis to measure the difference of compound feeds, there was significant difference in mean concentration of ZEN (*p* < 0.001). Cattle feeds were highly contaminated with ZEN compared to other feeds. Mean contamination levels of ZEN were 134.23 μg/kg in cattle feeds, 31.70 μg/kg in pig feeds, and 37.93 μg/kg in poultry feeds, with SD values of 134.83%, 36.44%, and 55.55%, respectively. The post-hoc test of each group was conducted according to the Scheffe method [15], and the detailed data are shown in Table 4.

**Figure 4:**
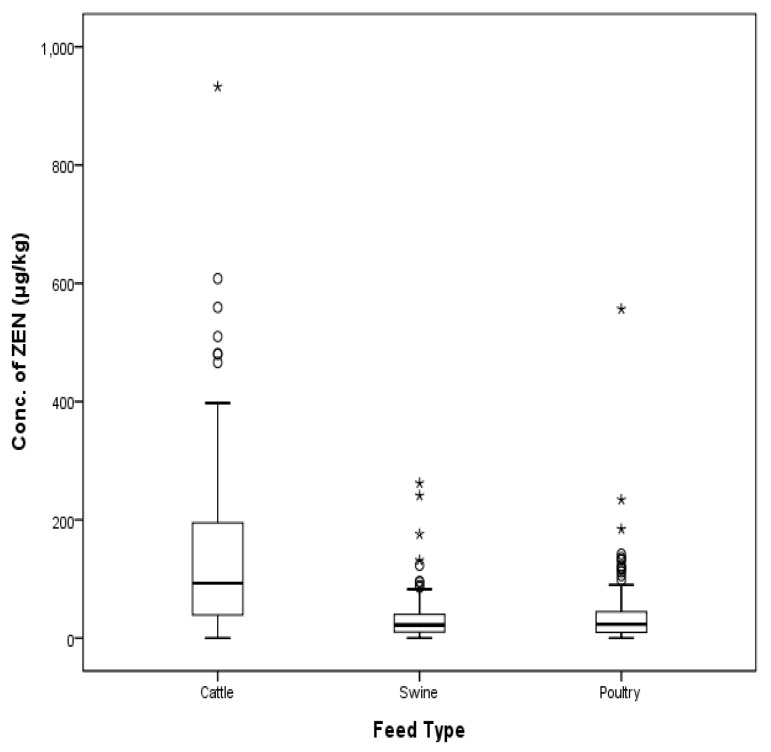
Distribution of ZEN in compound feeds for livestock (box-plot: whiskers at minimum and maximum, box at P25 and P75 with line at P50, °: values above the 75th percentile plus 1.5 times the inter-quartile distance, *: values above the 75th percentile plus 3.0 times the inter-quartile distance).

**Figure 5:**
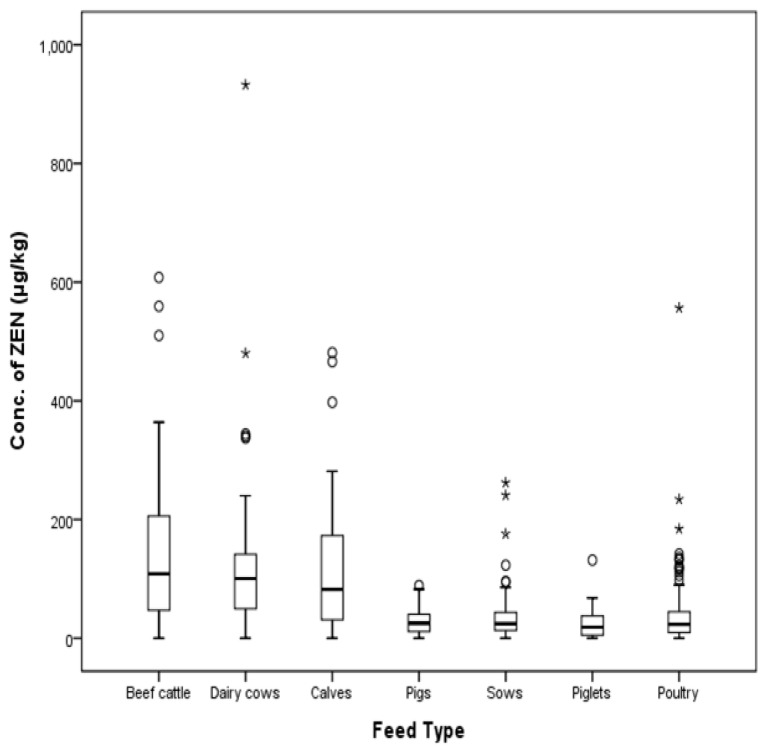
Distribution of ZEN in compound feed types (box-plot: whiskers at minimum and maximum, box at P25 and P75 with line at P50, °: values above the 75th percentile plus 1.5 times the inter-quartile distance, *: values above the 75th percentile plus 3.0 times the inter-quartile distance).

The EU and South Korea commission regulation for ZEN in calf feeds is 500 µg/kg, and the contamination level of ZEN in two beef cattle feeds (one lactating beef cattle feed, one high yielding dairy cow) was over the permissible limit. The level of ZEN in one compound feed out of 54 piglet samples also exceeded the EU commission regulation (100 µg/kg) with a level of 132 µg/kg. In sow and fattening pig feeds, the level of ZEN in one gestating sow feed (0.6%) exceeded the EU commission regulation (250 μg/kg) out of a total of 160 pig feed samples. Only four cases out of a total of 494 feed samples (1%) exhibited higher levels of ZEN than that listed in the EU commission regulation. The distribution of ZEN according to type of animal is shown in Figure 4 and Figure 5.

**Figure 6:**
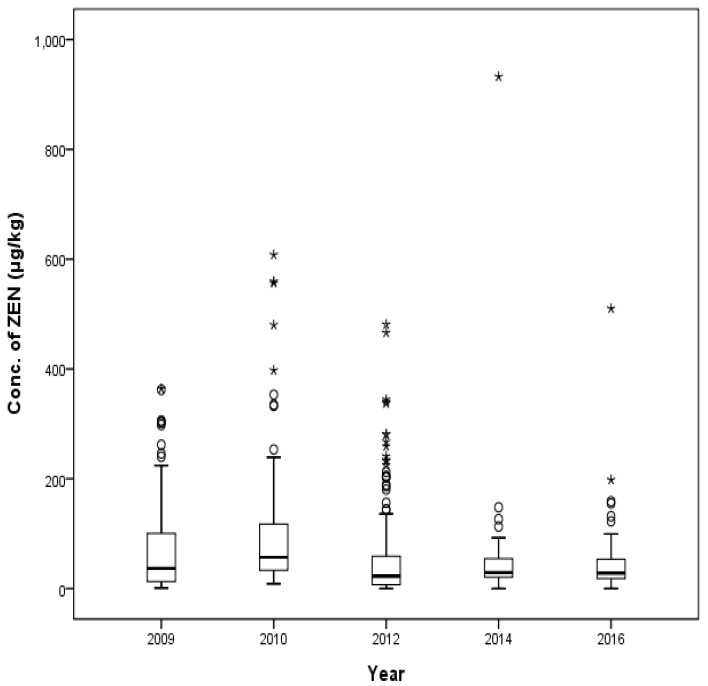
Distribution of ZEN in compound feed between 2009 and 2016 (box-plot: whiskers at minimum and maximum, box at P25 and P75 with line at P50, °: values above the 75th percentile plus 1.5 times the inter-quartile distance, *: values above the 75th percentile plus 3.0 times the inter-quartile distance).

A total of 494 compound feed samples (128, 90, 150, 69, and 66 animal feeds from the years 2009, 2010, 2012, 2014, and 2016, respectively) were checked for the distribution of ZEN (Figure 6). The median contamination level of ZEN in each year was 36.7 μg/kg, 56.8 μg/kg, 22.8 μg/kg, 29.1 μg/kg, and 28.0 μg/kg, respectively. Some early beef cattle feeds exhibited the maximum contamination levels in 2009 (364 μg/kg) and in 2010 (608 μg/kg). The detected level of ZEN in 2010 (608 μg/kg) exceeded the maximum ZEN levels in beef cattle feeds if we apply recent 2015 guidelines in Korea. High levels of ZEN were detected in middle beef calf feeds in 2012 (481 μg/kg), in high yielding dairy cow feeds in 2014 (932 μg/kg), and in lactating beef cattle in 2016 (510 μg/kg).

### 2.3. Occurrence of ZEN in Feed Ingredients between 2009 and 2016

A total of 159 feed ingredient samples were collected in 2009, 2010, 2012, 2014, or 2016, and their contamination levels were measured. The feed ingredients comprised 22 grains, 36 grain byproducts (brans), 76 meal products (vegetable proteins), 8 fibrous feeds, 13 food byproducts, and 4 other feed ingredients (beans, seed nuts, and mixed formulation), as shown in Table 5.

**Figure 7:**
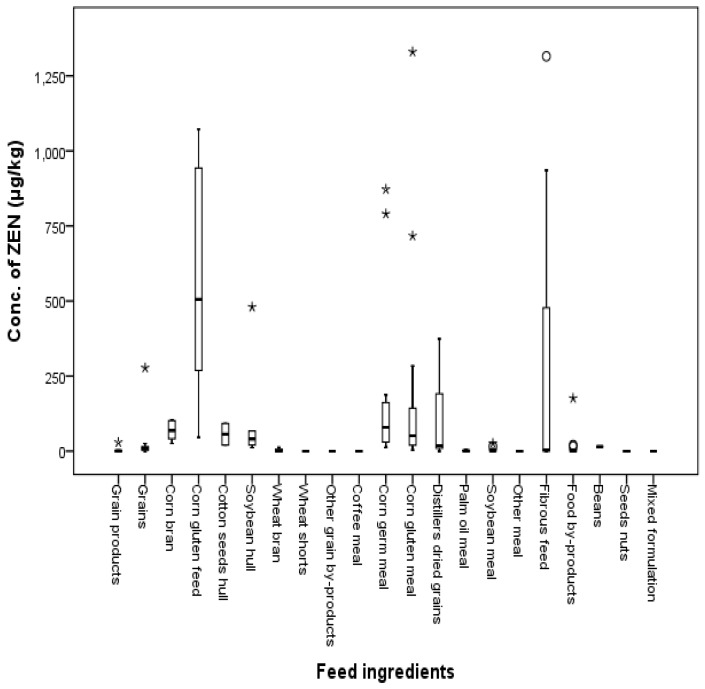
Distribution of ZEN in feed ingredients (box-plot: whiskers at minimum and maximum, box at P25 and P75 with line at P50, °: values above the 75th percentile plus 1.5 times the inter-quartile distance, *: values above the 75th percentile plus 3.0 times the inter-quartile distance).

**Figure 8:**
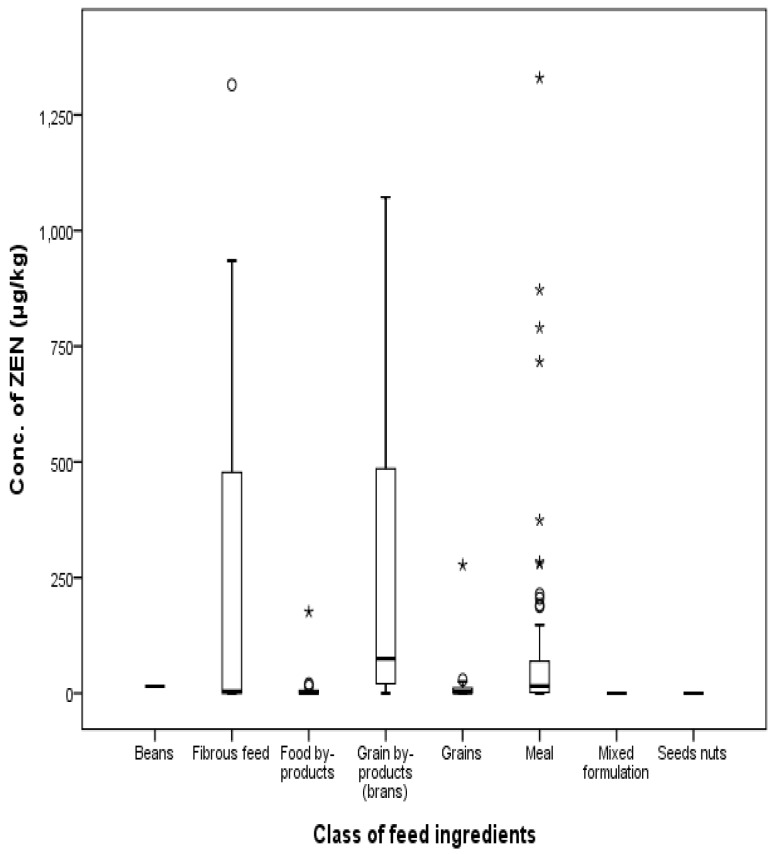
Distribution of ZEN in feed ingredients by class (box-plot: whiskers at minimum and maximum, box at P25 and P75 with line at P50, °: values above the 75th percentile plus 1.5 times the inter-quartile distance, *: values above the 75th percentile plus 3.0 times the inter-quartile distance).

The total incidence of ZEN contamination was 77%, 83%, 82%, 50%, 62%, and 100% in grains, grain byproducts, meal, fibrous feed, food byproducts, and beans, respectively. Overall, 77% of the total samples were determined to be contaminated with ZEN. The mean concentration of ZEN was 128.8 μg/kg, ranging from 1 to 1330 μg/kg. ZEN was detected in grains, grain byproducts, meal, fibrous feed, food byproducts, and beans at 19 μg/kg, 288 μg/kg, 95 μg/kg, 285 μg/kg, 18 μg/kg, and 15 μg/kg, respectively. The maximum concentration of ZEN was 277 μg/kg in grains, 1072 μg/kg in corn gluten feeds, 1330 μg/kg in corn gluten meals, 1315 μg/kg in fibrous feed, 176 μg/kg in food by-products, and 15 μg/kg in beans. ZEN was not detected in wheat shorts, other grain byproducts, coffee meal, other meals, seed nuts, or mixed formulation. Twenty-three percent of total feeds were contaminated with ZEN lower than LOD values. The distribution of ZEN by feed type is shown in Figure 7 and Figure 8.

Compared to the EU commission regulation of grains (2000 μg/kg) and the regulation of South Korea (3000 μg/kg), the maximum contamination level in Korea was 277 μg/kg; therefore, no feeds exceeded the commission regulation. Corn-gluten meals and corn-gluten feeds were contaminated with ZEN at levels of 1330 μg/kg and 1072 μg/kg, respectively.

**Figure 9:**
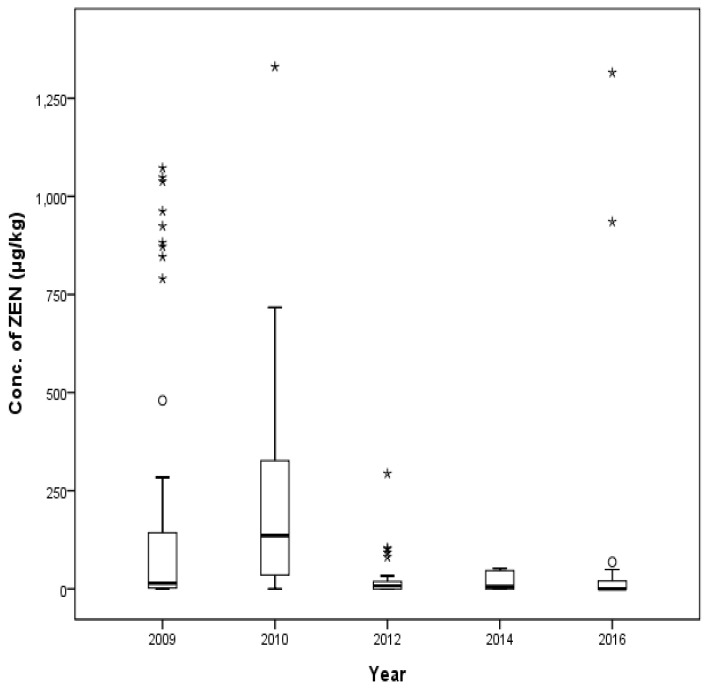
Distribution of ZEN in feed ingredients between 2009 and 2016 (box-plot: whiskers at minimum and maximum, box at P25 and P75 with line at P50, °: values above the 75th percentile plus 1.5 times the inter-quartile distance, *: values above the 75th percentile plus 3.0 times the inter-quartile distance).

The total of 159 feed ingredient samples included 66 (2009), 23 (2010), 30 (2012), 17 (2014), and 23 (2016) feed samples. The median value of ZEN in each year was 15 μg/kg, 136 μg/kg, 8 μg/kg, 6 μg/kg, and 0 μg/kg, respectively. High levels of ZEN were detected in corn-gluten feed in 2009 (1072 μg/kg) and 2012 (294 μg/kg), in corn-gluten meal in 2010 (1330 μg/kg) and 2014 (52 μg/kg), and in fibrous feed in 2016 (1315 μg/kg). The overall contamination level in 2010 was significantly higher than in other years (*p* < 0.01). The detailed results of one-way layout-dispersion analysis are shown in Table 6, and the occurrence of ZEN in each year is shown in Figure 9.

## 3. Discussion

Various studies have been performed worldwide to determine contamination levels of ZEN in compound feeds. In Germany, monitoring of ZEN data in 2008 for a total of 246 compound feeds showed that 38% of 69 sucking piglet feeds, 35% of 51 sow feeds, and 33% of 126 growing pig feeds were contaminated with ZEN [17]. The maximum level of ZEN was 100 μg/kg in sucking piglet feeds and sow feeds. One compound feed for growing pigs exhibited the highest contamination level (400 μg/kg), which exceeded the EU commission regulation (250 μg/kg). In Poland from 2006 to 2009, ZEN was detected in most compound feeds (72% to 100% out of 183 samples) [18]. The maximum contamination levels of ZEN in total feeds ranged from 44 to 229 μg/kg with a mean of 11 to 17 μg/kg in that report. Another study showed a similar contamination ratio of 91 to 100% from 428 compound feeds [19]. The level of ZEN contamination in compound feeds was estimated as 99% of 92 samples in the Republic of South Africa. Interestingly, 51% of 47 compound feeds were contaminated with ZEN over the limit of quantitation. The detection rate of ZEN in total feeds ranged from 30 to 610 μg/kg with a mean of 88 μg/kg. The cattle feeds were contaminated with ZEN with 123 μg/kg of maximum level and 72 μg/kg of mean level. The poultry feeds exhibited the maximum contamination level of 610 μg/kg and a mean contamination level of 100 μg/kg. None of the feeds exceeded the EU commission regulations [19]. Similarly, ZEN was detected in 89% of 53 chicken feeds with a mean of 55.6 μg/kg and ranged from 0 to 400 μg/kg in Kuwait [20]. ZEN was detected in 31.7% of compound feeds out of 180 samples with a mean value of 7.79 μg/kg (range of 2.1~29.3 μg/kg) in Turkey [8]. In Croatia, 93% of 30 growing pig feeds were contaminated with ZEN over a range from 8.93 to 866 μg/kg, and the mean contamination level was 184 μg/kg. Contamination levels of ZEN in five feed samples exceeded the EU commission regulation (250 μg/kg) [21]. In the Netherlands, ZEN was detected in 28% of 72 dairy cow feeds with a mean contamination level of 80 μg/kg and a maximum concentration of 363 μg/kg [22]. Three piglet feeds exceeded the EU commission regulation with levels ranging from 171 to 229 μg/kg in Poland [18]. According to monitoring research performed in Germany, Poland, and Croatia, the contamination level of ZEN in pig feeds should be regulated because pig feeds showed the highest concentration of ZEN that exceeded the EU commission regulations.

In this study, ZEN contamination level was estimated in 494 compound feed samples (174 cattle feeds, 160 pig feeds, and 160 poultry feeds) distributed in South Korea between 2009 and 2016 (2009, 2010, 2012, 2014, and 2016). Most of the tested compound feeds were contaminated with ZEN ranging from 1 to 932 μg/kg (a mean of 70 μg/kg). ZEN was detected in 89% of 54 piglet feeds distributed in South Korea, with one case (one weanling piglet feed) exceeding EU and South Korea regulation levels by 132 μg/kg. In growing pig feeds, ZEN was detected in 98% of 48 samples at a maximum contamination level of 89 μg/kg. The 98% of 58 sow compound feeds were contaminated with ZEN, and one feed sample for gestating sows showed maximum contamination concentrations of 262 μg/kg, which were higher than listed in the EU commission and South Korea regulations (250 μg/kg for sows compound feeds). In the case of beef compound feeds, three compounds including one lactating beef cattle feed in 2016 (510 μg/kg) and one high yielding dairy cow feed in 2014 (932 μg/kg) showed high ZEN contamination levels that were higher than those listed in the EU commission regulations (500 μg/kg for dairy cattle) and South Korea regulations (500 μg/kg for ruminant). The mean ZEN contamination level (70 μg/kg) ranged from 11 to 269 μg/kg in compound feeds distributed in Korea and was slightly higher than that of Poland and lower than that of the Republic of South Africa.

The contamination of ZEN in feed ingredients has also been reported in many other countries. Döll & Dänicke reported that 75% of corn feeds out of 95 samples were contaminated with ZEN in Germany. However, grains showed a much lower contamination ratio (24% out of 499 samples). There was no feed exhibiting higher ZEN contamination levels than the EU commission regulations [17]. Even though there were no case reports related to a higher contamination of ZEN in feed ingredients in Germany, some compound feeds such as sow feed showed relatively high ZEN contamination levels that were close to the EU commission regulations (100 μg/kg). Furthermore, a growing pig feed exhibited very high ZEN contamination levels (400 μg/kg) that exceed the EU commission regulations (250 μg/kg) despite the low contamination level of ZEN in feed ingredients in corn and grains [17]. In Kuwait, Beg et al. reported that most wheat bran, soybean meal, and corn feed ingredients were contaminated with ZEN, with a mean contamination level of 51 μg/kg and maximum contamination concentration of 99.6 μg/kg [20]. Research performed in the United Kingdom also showed that the detection frequency and mean contamination level of corn gluten meals were 20% and 50~480 μg/kg, respectively [23]. Also, it has been reported that ZEN was found in corn bran at 245.6 μg/kg in South Africa [24]. Interestingly, ZEN was not detected in corn from Brazil [25]. The incidence rate of ZEN contamination in feed ingredients was 92.3% in China [26,27] and 92% in Upper Egypt [28]. Njobeh et al. performed a study in the Republic of South Africa and reported that contamination level in cattle feeds was higher than that in feeds of other animals, but this difference in animal feed had no direct connection with the content of corn in each feed. The content of corn in pig and poultry feeds was higher relative to that in cattle feed [19].

In feed ingredients distributed in South Korea, 77% of the total samples including grains, grain byproducts, meal, fibrous feed, food byproducts, and others (beans, seeds nuts, and mixed formulation) were found to be contaminated, with incidence levels of 77%, 83%, 82%, 50%, 62%, and 25%, respectively. ZEN was detected in all 17 grains, but the contamination levels did not exceed the EU commission regulation (2000 μg/kg). Mean ZEN contamination level in grains was estimated as 19 μg/kg. Sixteen soybean meal samples were also contaminated with ZEN at a 5 μg/kg mean contamination level, which was lower than that of other countries. The mean contamination level of distillers dried grain was analyzed as 81 μg/kg, which was similar to the results of a previous study. The mean contamination level of feed ingredients related to corn was 324 μg/kg (2009), 270 μg/kg (2010), 67 μg/kg (2012), 46 μg/kg (2014), and 20 μg/kg (2016). In our previous report in 2014 [12], which was performed separately in other research project with different samples, similar results were found in the contamination levels of the ZEN in animal feeds. Most compound feeds for cattle were contaminated with this mycotoxin, and the highest level of ZEN contamination was 405 µg/kg in cattle fatting feeds.

The ZEN contamination level in feed needs to be compared over many years to assess the relevance of ZEN contamination between feed ingredients and compound feeds, and monitoring enough feed ingredient samples over long periods of time is recommended. The reason why incongruent ZEN levels were found in compound feeds despite using the same feed ingredients that did not exceed the EU commission regulations might be due to higher ZEN regulation standards in feed ingredients. If feed ingredients with high ZEN levels are used to produce compound feed, the level of ZEN in the resultant compound feed could exceed the regulation guideline because the guideline for ZEN in feed ingredients is much higher than that for compound feed.

## 4. Materials and Methods

### 4.1. Chemicals and Reagents

Zearalenone (ZEN, Sigma, St. Louis, MO, USA) was used as standards for the analysis. Acetonitrile and methanol were applied for the extraction of ZEN. Phosphate buffered saline (Sigma, St. Louis, MO, USA) was used for buffer solution. The EASI-EXTRACT ZEARALENONE kit (R-Biopharm^®^, Darmstadt, Germany) and ZearalaTest kit (Vicam^®^, Nixa, MO, USA) were used for the purification of ZEN. The Bond Elut Mycotoxin Cartridge (Varian^®^, Palo Alto, CA, USA) was applied for solid-phase extraction and purification. Standard solution of ZEN was diluted with a mixture of acetonitrile and water (acetonitrile:water = 75:25, *v*/*v*).

### 4.2. Sampling of Compound Feeds and Feed Ingredients

ZEN contamination levels were evaluated in 653 different animal feed samples (494 compound feeds and 159 feed ingredients) produced domestically from 2009 to 2016. The sample was randomly collected from feed factories in Korea. The detailed data of compound feeds and feed ingredients such as name of feed and number of samples is shown in Table 7 and Table 8. Feed samples were prepared according to the sampling guide in the code for the control of feeds (Food and Agriculture Organization (FAO)/World Health Organization (WHO), 2004) [29]. One kilogram of sample was collected randomly from every ton of feed ingredients or compound feeds. Four different collected samples were mixed together and divided into again four groups. Five hundred grams of the each divided sample were analyzed for ZEN contamination. The classification of compound feeds and feed ingredients is shown in the supplementary data (Tables S1–S5). All samples were kept at −70 °C for further analysis.

### 4.3. HPLC Analysis of ZEN

The level of ZEN in feed ingredients and compound feeds was analyzed according to the guideline of International Conference on Harmonisation (ICH) [30]. After addition of extraction solvent (100~125 mL 75% acetonitrile, 2 g sodium chloride, and 1 mL Tween 20) into 25 g of ground sample, ZEN was extracted and the extract was filtered through Whatman filter paper (No. 4) (GE Healthcare Life Science, Maidstone, Kent, UK). PBS solution (80 mL) was mixed with 20 mL of filtered extract solution, and the 4-fold diluted solution (25 mL) was loaded onto an Immuno Affinity Column (IAC, R-Biopharm^®^, Darmstadt, Germany) and eluted with 3~5 mL of methanol under gravity after washing of the IAC column with 20 mL of PBS or distilled water (DW). Eluted solution was entirely dried under a soft nitrogen steam at 40~50 °C, and the residue was re-dissolved with 1 mL of mobile phase solution (acetonitrile:methanol:water = 10:55:35, *v*/*v*/*v*) or 20% acetonitrile. After membrane filtration (0.22 μm pore size), the level of ZEN was measured by HPLC analysis using an Agilent 1100 series HPLC system (Santa Clara, CA, USA) including a degasser, auto sampler, diode array detector, and fluorescence detector with a ZORBAX Eclipse XDB-C18 column (Agilent, Santa Clara, CA, USA) (4.6 mm × 250 mm, 3 μm). ZEN was separated in HPLC for 20 min at a constant flow of 1 mL/min (30 °C) using FLD at 275 nm (excitation) and 450 nm (emission). The mobile phase consisted of A (DW) and B (acetonitrile) and was adjusted in gradient mode of separation. ZEN was detected 14.0 min after injection of 50 μL of samples into the HPLC system.

### 4.4. Identification of ZEN by Mass Spectrometry

Liquid chromatography-tandem mass spectrometry (API 4000, Applied Biosystems MDS SCIX, Foster, CA, USA) was used to determine the type of ZEN in the negative electrospray ionizaion (ESI-) acquisition. YMC-Pack pro C18 RS column (I.D. 3.0 mm × length 150 mm 3 μm particle size) was purchased from YMC (Kyoto, Japan) for the separation of ZEN in LC-MS/MS analysis under gradient conditions with a flow rate of 0.3 mL/min with the mobile phase consisting of distilled water (DW) containing 2 mM ammonium acetate and 0.1% formic acid, and acetonitrile containing 2 mM ammonium acetate and 0.1% formic acid. 10 micro liters of sample was injected into the HPLC and LC-MS/MS system. The retention time was 11.55 min in the LC-MS/MS analysis system. Detection and quantification of ZEN were performed in the Multiple Reaction Monitoring (MRM) mode. Parameters were 20 psi curtain gas, medium collision gas, −4500 ion-spray voltage, and 500 °C turbo gas temperature. The most abundant precursor ion (*m*/*z* 317.1 [M − H]^−^) of ZEN was selected from extracted ion chromatogram of standard ZEN at 75 ppm and it was compared with that of ZEN in sample. Two products ions (*m*/*z* 131.0, *m*/*z* 175.0) were further monitored in MS/MS analysis to confirm ZEN in standard and sample.

### 4.5. Method Validation

The HPLC method to analyze ZEN contents was validated by evaluation of LOD, LOQ, linearity, accuracy, and precision according to the ICH guideline [31]. The LOD was determined at the lowest amount of ZEN at which the signal to noise ratio was 3. The LOQ was calculated as the lowest amount of ZEN that could be quantitated by measuring the concentration exhibiting a signal to noise ratio of 10. The linearity was analyzed at 50, 100, 250, and 500 ng/mL by examining the correlation coefficient (*R*) and the linear regression line for the concentration variation. The regression coefficient (*R*^2^) was used to determine the acceptable data to the regression line. Accuracy was estimated by analyzing a known concentration from low to high concentration of ZEN (2.5, 25, 100 and 500 μg/kg) in samples (reference materials), and the measured value was compared with the true value. The results of accuracy test were expressed as percent recovery by the assay of known added amount of ZEN in repeated analysis. The degree of repeatability was expressed as precision in this analytical method. The percent relative standard deviation (%RSD) was the main parameter to calculate precision using a statistically significant number of samples.

## Figures and Tables

**Table 1:** Guidelines for mycotoxin levels in animal feed in the EU and South Korea.

Mycotoxins	Products Intended for Animal Feed	Guidance Value in mg/kg (ppm)	
		EU	Korea
Zearalenone	Feed material		
	-Cereals and cereal products with the exception of maize by-products	2	3
	-Maize by-products	3	3
	Complementary and complete feed stuffs		
	-Complementary and complete feed stuffs for piglets and gilts (young sows)	0.1	0.1
	-Complementary and complete feed stuffs for sows and fattening pigs	0.25	0.25
	-Complementary and complete feed stuffs for calves, dairy cattle, sheep (including lamb), and goats (including kids)	0.5	0.5
	-Complementary and complete feed stuffs for ruminants	-	0.5
	-Other complementary and complete feed stuffs	-	1

**Table 2:** Summary of the method validation study.

*R*^2^	LOD (µg/kg)	LOQ (µg/kg)	Recovery			
			Spiked Concentrations (µg/kg)	Mean Recovery (%)	SD	RSD (%)
0.999	0.1~3	0.3~8	2.5	111.2	10.4	9.4
			25	101.0	4.2	4.2
			100	96.0	12.9	12.6
			500	100.9	13.8	2.8

*R*^2^: Coefficient of determination, LOD: Limit of detection, LOQ: Limit of quantitation, SD: Standard deviation, RSD: Relative standard deviation.

**Table 3:** ZEN concentrations in various compound feed types.

Livestock	Feed Type	N ^(a)^	LC(%) ^(b)^	Mean (μg/kg)
Beef cattle	Early beef cattle	21	0	120.1
	Middle beef cattle	8	12.5	133.9
	Late beef cattle	20	0	145.9
	Gestating beef cattle	29	3.4	149.1
	Lactating beef cattle	2	0	269.5
Dairy cows	Dairy cow in early lactation	16	0	128.8
	Dairy cow in mid lactation	5	0	175.9
	Dairy cow on dry	4	0	136.5
	High yielding dairy cow	9	11.1	167.4
	Gestating dairy cow	5	0	99.8
Calves	Early beef calf	9	11.1	187.5
	Middle beef calf	11	0	129.5
	Early dairy calf	1	0	16.6
	Middle dairy calf	15	0	111.8
	Late dairy calf	13	0	112.9
	Middle breeding calf	1	0	21.2
	Late breeding calf	5	0	39.1
Pigs	Early growing pig	30	3.3	25.9
	Late growing pig	18	0	31.2
Sows	Gestating sow	32	3.1	48.5
	Lactating sow	25	0	32.5
	Breeding gilt	1	0	25.7
Piglets	Sucking piglet	8	25.0	10.9
	Weanling piglet	46	8.7	27.2
Poultry	Early layer chick	9	0	40.0
	Middle layer chick	22	4.5	34.9
	Late layer chick	8	0	55.7
	Early laying hens	27	0	72.5
	Middle laying hens	11	18.2	28.8
	Late laying hens	2	50.0	11.9
	Early broiler	37	5.4	28.4
	Late broiler	33	0	29.6
	Finishing broiler	2	0	23.3
	Breeding broiler	9	0	14.0

^(a)^ N: number of samples; ^(b)^ LC: percentage of left censored results. This value in this experiment is the percentage of data below LOD which exhibits where the observed data below LOD is located in the experiment [16].

**Table 4:** Concentration mean and differences of ZEN in compound feeds.

Mycotoxins	Livestock	Conc. of Mycotoxin (μg/kg)		F	*p*
		Mean	SD		
Zearalenone	Cattle	134.23 ^b^	134.83	71.287 ***	0.000
	Swine	31.70 ^a^	36.44		
	Poultry	37.93 ^a^	55.55		

^b^ > ^a^ = significant mean difference by Scheffe tests; *** *p* < 0.001; F: F-value.

**Table 5:** Concentrations of ZEN in feed ingredients.

Class	Feed Groups	N ^(a)^	LC (%) ^(b)^	Mean (μg/kg)
Grains	Grains	12	8.3	31.3
	Grain products	10	40.0	3.5
Grain byproducts (Brans)	Corn gluten feed	16	0	576.7
	Soybean hull	6	0	110.1
	Wheat shorts	2	100	0.0
	Cotton seeds hull	2	0	56.4
	Wheat bran	3	66.7	4.2
	Corn bran	5	0	68.2
	Other grain byproducts	2	100	0.0
Meal	Soybean meal	16	25.0	4.6
	Corn gluten meal	22	0	162.1
	Corn germ meal	12	0	200.9
	Distillers dried grains	14	14.3	81.4
	Coffee meal	1	100	0.0
	Palm oil meal	7	42.9	1.6
	Other meal	4	100.0	0.0
Fibrous feed	Fibrous feed	8	50.0	284.8
Food byproducts	Food byproducts	13	38.5	17.5
Beans	Beans	1	0	14.8
Seeds nuts	Seeds nuts	2	100	0.0
Mixed formulation	Mixed formulation	1	100	0.0

^(a)^ N: number of samples; ^(b)^ LC: percentage of left censored results.

**Table 6:** Concentration mean and differences of ZEN in feed ingredients across the years.

Mycotoxin	Year	Conc. of Mycotoxin (μg/kg)		F	*p*
		Mean	SD		
Zearalenone	2009	169.07	320.95	3.265 *	0.013
	2010	247.41	305.36		
	2012	28.68	59.08		
	2014	18.17	22.30		
	2016	107.21	326.71		

* *p* < 0.05, F: F-value.

**Table 7:** Compound feed samples between 2009 and 2016.

Livestock	Feed Type	No. of Samples					
		Total	2009	2010	2012	2014	2016
Beef cattle	Early beef cattle	21	9	2	6	2	2
	Middle beef cattle	8	-	3	-	2	3
	Late beef cattle	20	7	4	6	1	2
	Gestating beef cattle	29	13	7	5	2	2
	Lactating beef cattle	2	-	-	-	-	2
Dairy cows	Dairy cow in early lactation	16	6	2	6	1	1
	Dairy cow in mid lactation	5	2	2	-	1	-
	Dairy cow on dry	4	1	-	-	2	1
	High yielding dairy cow	9	1	-	5	2	1
	Gestating dairy cow	5	-	3	-	1	1
Calves	Early beef calf	9	-	-	5	1	3
	Middle beef calf	11	1	-	6	1	3
	Early dairy calf	1	-	-	-	-	1
	Middle dairy calf	15	4	3	6	1	1
	Late dairy calf	13	3	4	5	1	-
	Middle breeding calf	1	-	-	-	-	1
	Late breeding calf	5	1	-	-	2	2
Pigs	Early growing pig	30	12	5	10	3	-
	Late growing pig	18	4	5	5	4	-
Sows	Gestating sow	32	6	10	10	3	3
	Lactating sow	25	5	5	10	3	2
	Breeding gilt	1	-	-	-	1	-
Piglets	Sucking piglet	8	-	-	5	2	1
	Weanling piglet	46	13	5	10	4	14
Poultry	Early layer chick	9	3	3	-	1	2
	Middle layer chick	22	4	7	6	3	2
	Late layer chick	8	-	-	5	3	-
	Early laying hens	27	5	6	10	3	3
	Middle laying hens	11	-	-	5	3	3
	Late laying hens	2	-	-	-	-	2
	Early broiler	37	12	7	10	4	4
	Late broiler	33	12	7	9	3	2
	Finishing broiler	2	-	-	-	-	2
	Breeding broiler	9	4	-	5	-	-
Total		494	128	90	150	60	66

**Table 8:** Feed ingredient samples between 2009 and 2016.

Class	Feed Type	No. of Samples					
		Total	2009	2010	2012	2014	2016
Grain	Grain	12	7	4	-	-	1
	Grain products	10	7	-	2	-	1
Grain-by products (Bran)	Corn gluten feed	16	9	5	2	-	-
	Soybean hull	6	5	-	-	1	-
	Wheat shorts	2	-	-	1	1	-
	Cotton seeds hull	2	-	-	1	1	-
	Wheat bran	3	-	-	2	1	-
	Corn bran	5	1	-	2	1	1
	Other grain by-products	2	-	-	-	1	1
Meal (Vegetable proteins)	Soybean meal	16	10	-	2	3	1
	Corn gluten meal	22	10	5	4	3	-
	Corn germ meal	12	4	4	2	1	1
	Distillers dried grains	14	2	5	6	-	1
	Coffee meal	1	-	-	1	-	-
	Palm oil meal	7	4	-	3	-	-
	Other meal	4	-	-	-	1	3
Fibrous feed	Fibrous feed	8	-	-	-	2	6
Food by-products	Food by-products	13	7	-	2	1	3
Beans	Beans	1	-	-	-	-	1
Seeds nuts	Seeds nuts	2	-	-	-	-	2
Mixed formulation	Mixed formulation	1	-	-	-	-	1
Total		159	66	23	30	17	23

## Supplementary Materials

The following are available online at www.mdpi.com/2072-6651/9/7/223/s1, Table S1. Classification of compound feeds for cattle, Table S2. Classification of compound feeds for swine, Table S3. Classification of compound feeds for poultry, Table S4. Classification of compound feeds for dairy cows, Table S5. Classification of feed ingredients.
