# Supplementary material for: The Occurrence of Zearalenone in South Korean Feedstuffs between 2009 and 2016
Source: Toxins (Basel). 2017 Jul 15;9(7):223. doi: 10.3390/toxins9070223 (PMC5535170; doi:10.3390/toxins9070223)
Supplement: Supplementary file 1 [file toxins-09-00223-s001.pdf]

Supplementary Materials: The Occurrence of Zearalenone in South Korean Feedstuffs between 2009 and 2016

Hansub Chang, Woori Kim, Ju-Hee Park, Dongho Kim, Choong-Ryeol Kim, Soohyun Chung and Chan Lee

Table S1. Classification of compound feeds for cattle.

Feed Type	Period for feeding
Early breeding calf	Birth to 3 months
Middle breeding calf	3 to 6 months
Late breeding calf	6 to 12 months
Sire or breeding bull	After 13 months
Gestating beef	13 months to farrowing
Lactating beef	Farrowing to 3 months after birth
Early beef calf	Before 3 months
Middle beef calf	After 3 months to 250 kg of body weight
Early beef cattle	250 kg to 400 kg of body weight
Middle beef cattle	400 kg to 500 kg of body weight
Late beef cattle	Over 500 kg of body weight

Table S2. Classification of compound feeds for swine.

Feed Type	Period for feeding
Sucking piglet	Before lactating
Weanling piglet	Over 5 kg of b.w. or after lactating to 20 kg of b.w.
Early growing pig	20 kg to 50 kg of b.w.
Late growing pig	50 kg to 80 kg of b.w.
Growing pig	50 kg or 80 kg of b.w. to before the 15th for shipment
Growing-finishing pig	Before the 15th for shipment to shipment
Sire, boar	Over 25 kg of b.w.
Gilt	25 kg of b.w. to before pregnant
Gestating sow	Gestating period
Lactating sow	Lactating period

Table S3. Classification of compound feeds for poultry.

Feed Type	Period for feeding
Early layer chicks	Before 6 to 10 weeks
Middle layer chicks	6 to 12 weeks or 2 weeks before laying
Late layer chicks	12 weeks to 2 weeks before laying
Early broiler chicks	3 weeks or before 6 weeks
Middle broiler chicks	3 weeks or 6 weeks to before laying

Before laying	2 weeks before laying to laying commencement
Early laying	Laying commencement to 40 weeks
Middle laying	40 weeks to 65 weeks
Late laying	After 65 weeks
Breeding broiler	Layer breeder or broiler breeder
Early broiler	Before 3 weeks
Middle broiler	3 weeks to before the 7~10th before shipment
Finishing broiler	After the 7~10th before shipment to shipment

Table S4. Classification of compound feeds for dairy cows.

Feed Type	Period for feeding
Early dairy calf	Birth to 3 months
Middle dairy calf	3 months to 6 months
Late dairy calf	6 months to before pregnancy
Gestating dairy cow	Pregnancy to 2 months after farrowing
Dairy sires	After 13 months
Dairy cow in early lactation	After farrowing to lactation 3 months (31 to 40 kg of milk production)
Dairy cow in mid lactation	Lactation 3 months to 6 months (21~30 kg of milk production)
Dairy cow in late lactation	Lactation 6 months to dry period (11~20 kg of milk production)
Dairy cow on dry	Dry period (Less than 10 kg of milk production)
High yielding dairy cow	Over 40 kg of milk production

Table S5. Classification of feed ingredients.

Class	Feed type
Grains	Grains
	Grain products
Grain byproducts (Bran)	Corn gluten feed
	Soybean hull
	Wheat shorts
	Cotton seeds hull
	Wheat bran
	Corn bran
	Other grain byproducts
Meal (Vegetable proteins)	Soybean meal
	Wheat gluten
	Corn gluten meal
	Corn germ meal
	Distillers dried grains
	Coffee meal

	Palm oil meal
	Other meal
Fibrous feed	Fibrous feed
Food byproducts	Food byproducts
Beans	Beans
Seed nuts	Seed nuts
Mixed formulation	Mixed formulation
